# Oxidative Stress Markers and Na,K-ATPase Enzyme Kinetics Are Altered in the Cerebellum of Zucker Diabetic Fatty fa/fa Rats: A Comparison with Lean fa/+ and Wistar Rats

**DOI:** 10.3390/biology13100759

**Published:** 2024-09-25

**Authors:** Dominika Radosinska, Alexandra Gaal Kovalcikova, Roman Gardlik, Maria Chomova, Denisa Snurikova, Jana Radosinska, Norbert Vrbjar

**Affiliations:** 1 Institute of Medical Biology, Genetics and Clinical Genetics, Faculty of Medicine, Comenius University in Bratislava, Sasinkova 4, 811 08 Bratislava, Slovakia; dominika.radosinska@fmed.uniba.sk; 2 Department of Pediatrics, National Institute of Children’s Diseases, Faculty of Medicine, Comenius University in Bratislava, 833 40 Bratislava, Slovakia; alexandra.gaal.kovalcikova@fmed.uniba.sk; 3 Institute of Molecular Biomedicine, Faculty of Medicine, Comenius University in Bratislava, Sasinkova 4, 811 08 Bratislava, Slovakia; roman.gardlik@imbm.sk; 4 Institute of Medical Chemistry, Biochemistry and Clinical Biochemistry, Faculty of Medicine, Comenius University in Bratislava, Sasinkova 2, 813 72 Bratislava, Slovakia; maria.chomova@fmed.uniba.sk; 5 Centre of Experimental Medicine, Slovak Academy of Sciences, Institute for Heart Research, Dúbravská Cesta 9, 841 04 Bratislava, Slovakia; usrddena@savba.sk (D.S.); norbert.vrbjar@savba.sk (N.V.); 6 Institute of Physiology, Faculty of Medicine, Comenius University in Bratislava, Sasinkova 2, 811 08 Bratislava, Slovakia

**Keywords:** Na,K-ATPase enzyme, oxidative stress, diabetes severity, cerebellum, Zucker diabetic fatty rats

## Abstract

**Simple Summary:**

Type 2 diabetes mellitus (T2DM) is a global health burden that adversely affects various organs, including the brain, leading to neurodegeneration. Recent research emphasizes the cerebellum’s role in studying the interactions between T2DM, obesity, aging, and brain energy metabolism. This study focused on the cerebellum of Zucker diabetic fatty (ZDF) rats, a model that mirrors the diversity of T2DM in humans. The primary objective was to measure oxidative stress markers and kinetic properties of sodium–potassium ATPase (Na,K-ATPase) in relation to T2DM severity (not documented yet). Na,K-ATPase is an active transport mechanism crucial for maintaining unequal cation distributions across the plasma membrane, and its activity has been shown to be altered by diabetes in various organs. While oxidative stress is well established in the pathogenesis of diabetes, this study confirmed systemic oxidative and carbonyl damage in ZDF rats and provided new evidence of such damage in cerebellar tissue. However, no differences were found in cerebellar oxidative stress markers based on T2DM severity. Additionally, Na,K-ATPase activity was higher in the cerebellum of ZDF rats compared with controls, suggesting the presence of compensatory mechanisms in this brain region in aged ZDF animals. However, further research is needed to confirm and elucidate this phenomenon.

**Abstract:**

Type 2 diabetes mellitus has been referred to as being closely related to oxidative stress, which may affect brain functions and brain glucose metabolism due to its high metabolic activity and lipid-rich content. Na,K-ATPase is an essential enzyme maintaining intracellular homeostasis, with properties that can sensitively mirror various pathophysiological conditions such as diabetes. The goal of this study was to determine oxidative stress markers as well as Na,K-ATPase activities in the cerebellum of Zucker diabetic fatty (ZDF) rats depending on diabetes severity. The following groups of male rats were used: Wistar, ZDF Lean (fa/+), and ZDF (fa/fa) rats, arbitrarily divided according to glycemia into ZDF obese (ZO, less severe diabetes) and ZDF diabetic (ZOD, advanced diabetes) groups. In addition to basic biometry and biochemistry, oxidative stress markers were assessed in plasma and cerebellar tissues. The Na, K-ATPase enzyme activity was measured at varying ATP substrate concentrations. The results indicate significant differences in basic biometric and biochemical parameters within all the studied groups. Furthermore, oxidative damage was greater in the cerebellum of both ZDF (fa/fa) groups compared with the controls. Interestingly, Na,K-ATPase enzyme activity was highest to lowest in the following order: ZOD > ZO > Wistar > ZDF lean rats. In conclusion, an increase in systemic oxidative stress resulting from diabetic conditions has a significant impact on the cerebellar tissue independently of diabetes severity. The increased cerebellar Na,K-ATPase activity may reflect compensatory mechanisms in aged ZDF (fa/fa) animals, rather than indicating cerebellar neurodegeneration: a phenomenon that warrants further investigation.

## 1. Introduction

In recent years, diabetes mellitus (DM), characterized by chronic hyperglycemia, has reached epidemic proportions. The number of people with DM is projected to reach approximately 642 million by 2040 [1]. Type 2 diabetes mellitus (T2DM) accounts for approximately 90% of all diabetes cases. Its pathophysiology is characterized by insulin dysregulation, leading to insulin resistance and pancreatic β-cell dysfunction, which primarily affects the liver, muscles, and adipose tissue [2]. These disruptions contribute to macrovascular and microvascular complications in various organs, including the heart, kidneys, eyes, and brain. Additionally, increasing evidence links T2DM with cognitive impairment and neurodegeneration, resulting in structural brain changes [3,4].

Increased free radical production due to high blood glucose levels can lead to lipid peroxidation, protein carbonylation, and DNA damage, creating a link between metabolic disorders and neuropathology. This oxidative and carbonyl damage to various biomolecules is also associated with inflammation [5]. Both oxidative stress and brain inflammation are implicated in DM and neurodegenerative diseases such as Alzheimer’s disease, often referred to as “type 3 DM”. Despite the complex and unclear etiology of both diseases, they share similar risk factors and pathophysiological mechanisms [4]. The exact cause of structural and functional changes in the diabetic brain remains unclear. While most studies have focused on the cerebrum, increasing evidence suggests that the cerebellum, with its extensive functional connectivity, is also involved in the neuropathology of cognitive dysfunction in T2DM [6,7,8].

The sodium–potassium ATPase (Na,K-ATPase), as a large transmembrane protein, represents the active transport mechanism responsible for the maintenance of resting membrane potential in all eukaryotic cells of the organism, as well as its recovery in excitable cells. This enzyme activity was shown to be modified in DM in different organs [9,10]. Furthermore, the Na,K-ATPase enzyme has been found to be associated with oxidative stress in Alzheimer’s disease and to interact with amyloid β peptides [11,12].

Animal studies, including those involving genetically Zucker diabetic fatty (ZDF) rats, also indicate a link between DM and neurodegeneration [13]. This model features a genetic mutation (fa) that leads to a truncated leptin receptor, resulting in adult-onset impaired glucose tolerance and obesity [14]. For this model induced by alteration in a single gene, separate subpopulations differing in various phenotypic effects like body weight, insulin level, glycemia, vascular, and neural system variations were identified [15]. In multiple respects, the ZDF rats reflect the diversity seen in humans suffering from T2DM [16]. To investigate the impact of elevated glucose levels on brain pathology, we used ZDF (fa/fa) rats, an inbred model for T2DM. The goal of this study was to determine markers of oxidative and carbonyl stress, as well as Na,K-ATPase activities in the cerebellum of ZDF rats depending on diabetes severity. The working hypothesis was that more advanced diabetes would lead to greater oxidative damage to cerebellar tissue and further impairment of Na,K-ATPase activities. In addition, this study focused on differences between two control rat strains commonly used in experiments: lean ZDF fa/+ rats, which serve as standard controls for the ZDF fa/fa rats, and Wistar rats, an outbred strain of a generally accepted model of albino rats with multiple uses in research.

## 2. Materials and Methods

### 2.1. Experimental Model

Laboratory rats were sourced from the breeding facility at the Department of Toxicology and Laboratory Animal Breeding, Centre of Experimental Medicine, Slovak Academy of Sciences, Dobra Voda, Slovak Republic. We studied four groups of male rats: Wistar (W), as an absolute control; ZDF Lean (ZL), as a standard control; ZDF rats with lower glycemia (ZO) below 10 mmol·L^−1^; and ZDF rats with developed hyperglycemia (ZOD) above 10 mmol·L^−1^. The ZDF rats were categorized into two subgroups according to fasting glycemia at the 36th week of age, while this splitting was performed arbitrarily with a cut-off value of 10 mmol·L^−1^ as published previously [9,17]. All the laboratory animals were genotyped for the leptin gene receptor as described previously [9,17]. The ZDF rats displayed the fa/fa genotype, while the control ZDF lean rats exhibited heterozygosity (fa/+) and the W rats showed a wild-type genotype (+/+). All rats were kept in a 12 h light, 12 h dark cycle, at a constant temperature of 20–22 °C, with access to water and food ad libitum. From the 3rd to 7th week of life, they were fed a standard pellet diet, and from the 8th week onward, they were fed Purina Rodent LabDiet 5008 (LabDiet, London, UK). Between the 38th and 39th week of life, the rats were decapitated in non-fasting conditions. Their blood was collected into K_3_EDTA tubes and centrifuged (850× *g*, 10 min at 4 °C), and plasma was separated. Tissues were rapidly excised, weighed, and stored at −80 °C for further analyses.

Body weight, fasting glucose, and insulin levels were assessed at 36 weeks of age in all rats (Table 1). Homeostatic model assessment (HOMA) indexes for insulin sensitivity (HOMA-IS), insulin resistance (HOMA-IR), and β-cell function (HOMA-β) were calculated following the method described previously [17]. The HOMA-IR index indicates the requirement for insulin release from the pancreas to maintain normoglycemia, while the HOMA-IS index reflects cellular responsiveness to insulin. The HOMA-β index serves as a measure of insulin secretion, derived from fasting plasma glucose and insulin concentrations [18].

All protocols were approved by the Department of Animal Wellness, State Veterinary, and Food Administration of the Slovak Republic (decision no. Ro-493/18-221/3) and executed in compliance with the regulations outlined by the State Veterinary and Food Administration of the Slovak Republic. Additionally, the procedures were approved by the Ethical Committee of the Institute of Pharmacology and Institute of Medical Chemistry, Biochemistry, and Clinical Biochemistry, adhering to the guidelines set forth by the European Convention for the Protection of Vertebrate Animals used for Experimental and other Scientific Purposes, Directive 2010/63/EU of the European Parliament.

### 2.2. Determination of Parameters of Oxidative Stress in Plasma and Cerebellar Tissue

Selected parameters of oxidative stress and markers of antioxidant status were evaluated by spectrophotometric and fluorescent methods in plasma samples and 10% homogenate of cerebellar tissues according to the methods described in more detail previously [17].

Estimation of lipid peroxidation involved measuring thiobarbituric acid reactive substances (TBARSs) with standards (1,1,3,3-tetraethoxypropane). For quantification, the fluorescence signal was measured (λ_ex_ = 515 nm, λ_em_ = 553 nm). To evaluate protein oxidative damage, advanced oxidation protein products (AOPPs) were estimated spectrophotometrically (λ = 340 nm). Fructosamine, an early glycation marker of proteins, was determined by mixing samples and standards with nitro blue tetrazolium. After incubation and absorbance measurement (λ = 530 nm), fructosamine levels were quantified. Advanced glycation end product-associated fluorescence (AGE-Fl) was assessed as a marker of carbonyl stress. Samples mixed with phosphate-buffered saline were measured for autofluorescence (λ_ex_ = 370 nm, λ_em_ = 440 nm). Ferric reducing antioxidant power (FRAP) assessed antioxidant status. Pre-warmed FRAP reagent containing acetate buffer, tripyridyl-s-triazine, FeCl_3_·6H_2_O, and distilled water was added to samples and standards (100 mmol·L^−1^ FeSO_4_·7H_2_O). After incubation and mixing, absorbance was measured (λ = 593 nm). The reduced-to-oxidized-glutathione ratio (GSH/GSSG) served as a marker of general redox balance. For reduced glutathione, samples and standards were mixed with O-phtaldehyde solution and phosphate-buffered saline. After incubation, fluorescence was measured (λ_ex_ = 350 nm, λ_em_ = 460 nm). For oxidized glutathione, samples and standards mixed with N-ethylmaleimide were incubated, then pipetted into a new multi-well plate with O-phtalaldehyde solution and NaOH. Following the next incubation and mixing, fluorescence was measured (λ_ex_ = 350 nm, λ_em_ = 460 nm).

### 2.3. Isolation of Plasma Membrane Fraction of Cerebellum

The membrane fraction of the cerebellar tissue containing the Na,K-ATPase was prepared by the method of Jorgensen [19]. For determination of protein concentration, the method of Lowry et al. [20] was utilized.

### 2.4. Kinetic Parameters of Na,K-ATPase Enzyme

Samples containing 20 μg·mL^−1^ of membrane proteins were diluted in an incubation reaction buffer and pre-incubated for 20 min at 37 °C without substrate. The activity of Na,K-ATPase in the cerebellar tissue was assessed by varying concentrations of ATP substrate (0.16–8 mmol·L^−1^) for another 20 min as described previously [9]. Following this incubation period, the chemical reaction was stopped by adding 0.3 mL of a 12% trichloroacetic acid solution. Subsequently, the liberated inorganic phosphorus resulting from ATP hydrolysis reacted with ammonium molybdate, which is then measured spectrophotometrically at λ = 700 nm.

The kinetic parameters V_max_ (the maximum velocity of enzyme reaction) and K_m_ (the substrate ATP concentration required for half-maximal enzyme activation) were determined from the data using direct nonlinear regression based on the Michaelis–Menten equation. The V_max_ parameter reflects the quantity of active Na,K-ATPase molecules, while K_m_ represents the enzyme’s affinity to the substrate.

### 2.5. Statistical Analyses

The obtained data are expressed as means ± standard errors of the mean. Their normal distribution was assessed by the use of the D’Agostino–Pearson test. One-way analysis of variance (ANOVA) with Tukey’s post hoc test was employed to discern differences among all groups. Graphical representations and statistical analyses were conducted using statistical software SigmaPlot 13 and GraphPad Prism 7.

## 3. Results

### 3.1. Main Characteristics of the Laboratory Rats

The general characteristics of rats are presented in Table 1. The rats in the ZL group had a lower body weight (BW) than those in the W group (*p* = 0.019), the ZO group (*p* < 0.0001), and the ZOD group (*p* = 0.0008). Additionally, the W rats had a lower BW compared with the ZO rats (*p* < 0.0001), and the ZOD group had a lower BW compared with the ZO group (*p* = 0.0095). The brain weight was highest in the W group compared with the ZL (*p* = 0.04), ZO (*p* = 0.0002), and ZOD (*p* = 0.0002) groups. A similar trend was observed for the cerebellum weight (CW) with W rats having higher CW than the ZL (*p* = 0.03), ZO (*p* = 0.005), and ZOD (*p* = 0.005) rats. The CW/BW ratio was greater in both controls compared with both groups of ZDF fa/fa rats.

The highest fasting blood glucose levels were observed in the ZOD rats, which were significantly higher than those in the W, ZL, and ZO groups (all *p* < 0.0001). Additionally, the ZO rats had higher glycemia compared with the ZL rats (*p* = 0.02). Plasma insulin measurements showed the highest concentration in the ZO group compared with all other groups (W: *p* < 0.0001; ZL: *p* < 0.0001; ZOD: *p* = 0.0001), while the ZL group had the lowest concentration (W: *p* = 0.007; ZOD: *p* = 0.006). The HOMA-IR index was significantly higher in both ZDF fa/fa groups compared with both controls (both *p* < 0.0001). Furthermore, the ZL rats had a lower HOMA-IR than the W rats (*p* = 0.0043). Conversely, the HOMA-IS index was lower in both groups of ZDF fa/fa rats compared with the control groups (W: *p* = 0.024; ZL: *p* < 0.0001), with the ZL rats showing the highest value of HOMA-IS value among all groups. The HOMA-β index, indicating beta cell function, was significantly higher in the ZO rats compared with both control groups (W: *p* < 0.001; ZL: *p* < 0.0001). Additionally, the ZL rats had lower HOMA-β values than the W rats (*p* = 0.0016), and the ZOD rats had a lower index compared with the ZO rats (*p* < 0.0001).

### 3.2. Parameters of Oxidative Stress and Antioxidant Status in Blood Plasma and Cerebellum

In their plasma, both ZDF fa/fa groups had statistically higher levels of oxidative stress markers—the TBARS, AOPP, and the early carbonyl stress marker—fructosamine, compared with both controls (Table 2). However, in the cerebellum, this trend was observed only for the TBARS marker. The AOPP marker was elevated in both ZDF fa/fa groups compared with the W rats (ZO: *p* = 0.005; ZOD: *p* = 0.02). The fructosamine level was higher in the ZO group compared with both controls (both *p* = 0.004). Additionally, the AGE-Fl, an advanced glycation parameter, was significantly higher in both ZDF fa/fa groups than in both control groups in the cerebellum.

The antioxidant parameter FRAP was greater in both ZDF fa/fa groups compared with both control groups in plasma samples. In cerebellar tissues, the ZO rats had significantly higher FRAP values than the W rats (*p* = 0.034); additionally, the ZL rats had higher FRAP values than the W rats (*p* = 0.038). The general marker of oxidative stress, the GSH/GSSG ratio, was the lowest in plasma from ZO rats compared with both controls and in cerebellar tissue; it was the lowest value in the ZOD group compared with the W group (*p* = 0.013). Furthermore, the selected oxidative stress parameters were generally significantly different between the ZO and ZOD rats in plasma, but not in the cerebellum, as are presented in Table 2.

### 3.3. Na,K-ATPase Enzyme Kinetic in the Cerebellum

**Figure 1:**
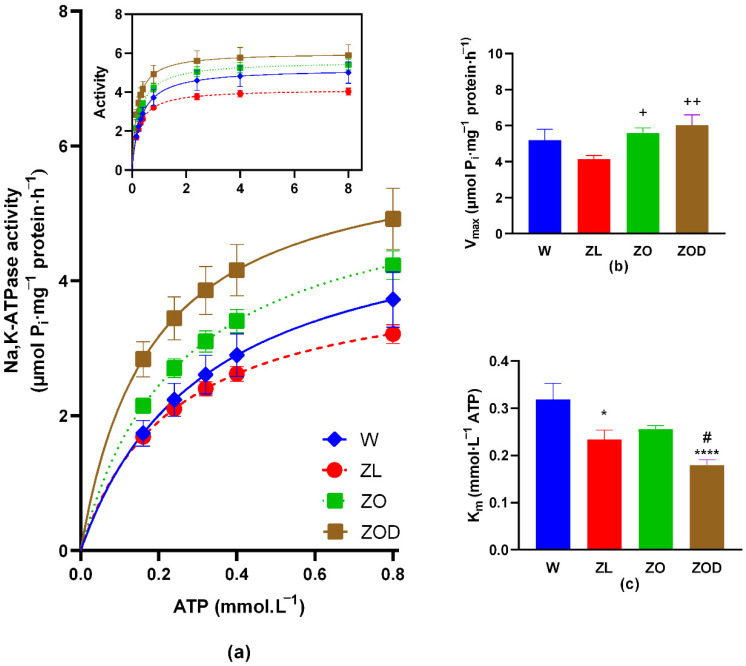
Na,K-ATPase enzyme kinetics. (**a**) Activity of Na,K-ATPase enzyme in presence of ATP substrate at low concentrations (range: 0.16–0.8 mmol·L⁻^1^). Inset: activity of the enzyme in the whole examined range of ATP. (**b**) Values of V_max_ in all experimental groups. (**c**) Values of K_m_ in all experimental groups. Abbreviation: W—Wistar, ZL—lean fa/+, Zucker diabetic fatty (ZDF) fa/fa rats divided into ZO rats with lower glycemia (<10 mmol·L^−1^) and ZOD rats with higher glycemia (>10 mmol·L^−1^). Data are presented as means ± standard errors of mean (SEM). * *p* < 0.05, **** *p* < 0.0001 vs. W; ^+^ *p* < 0.05, ^++^ *p* < 0.01 vs. ZL; ^#^ *p* < 0.05 vs. ZO.

Activation of cerebellar Na,K-ATPase by its substrate resulted in increased enzyme activity across the examined ATP concentration range in both ZDF (fa/fa) groups compared with control groups, with the most pronounced effect observed in the ZOD rats. At the lowest concentration studied (0.16 mmol·L⁻^1^), Na,K-ATPase activity in the ZOD group was increased by 63%, 68%, and 32% compared with the W, ZL, and ZO groups, respectively. However, as the substrate concentration increased, the disparity in enzyme activity diminished, except in the ZL group, where differences compared with the ZO and W groups widened. At the highest concentration of ATP substrate (8.00 mmol·L⁻^1^), Na,K-ATPase activity in the ZOD rats was increased by 18%, 46%, and 9% compared with the W, ZL, and ZO rats, respectively (Figure 1a).

V_max_ values were significantly higher in both ZDF fa/fa groups, notably higher than in the ZL rats (ZO: 35%, ZOD: 45%). Nonetheless, K_m_ values were significantly lower in the ZL (by 27%) and ZOD (by 44%) rats compared with the W rats, and also lower in the ZOD rats (by 30%) compared with the ZO rats (Figure 1b,c).

## 4. Discussion

Recent research highlights the importance of the cerebellum in studying the interplay between T2DM, hyperglycemia, hypo/hyperinsulinemia, obesity, aging, and brain energy metabolism [21,22,23]. This study is the first to demonstrate changes in oxidative stress markers and Na,K-ATPase activity in the cerebellum of the ZDF rats based on the severity of diabetes. The ZDF fa/fa rats were categorized according to their glucose, insulin, and HOMA indexes. The ZO rats exhibited lower glucose levels but the highest insulin levels among all groups, indicating phenotypic obesity with insulin resistance and a less developed stage of diabetes, consistent with previous findings [17,24]. Conversely, the highest glucose levels observed in the ZOD rats, along with lower insulin levels compared with the ZO rats, indicate fully developed diabetes, corroborating earlier observations [16]. These findings were supported by the HOMA indexes: HOMA-IR was elevated in both ZDF groups, while HOMA-IS was lower. The HOMA-β index, which estimates pancreatic β-cell function, was reduced in the ZOD rats, reflecting a progressive decline in β-cell function and consequent hyperglycemia. In contrast, the ZO group exhibited an increased HOMA-β index, suggesting a compensatory mechanism for insulin resistance as previously documented [25,26]. The decrease in the HOMA-β index, combined with low HOMA-IR and high HOMA-IS indexes in the ZL rats, likely indicates that low insulin concentrations are sufficient for maintaining normoglycemia due to the high insulin sensitivity in these animals. The observation of significantly lower body weight in the ZOD rats than the ZO ones in the age range of 38–39 weeks is in agreement with data published previously for 40-week-old ZDF rats compared with Zucker non-diabetic fatty rats [15].

Based on diabetes severity estimated by hyperglycemia, insulin resistance, and β-cell dysfunction, this study provides data regarding the markers of oxidative stress in plasma (systemic level) and cerebellar tissue. It was shown that a high-fat diet combined with a high-sucrose diet for 21 weeks induced an increase in systemic oxidative stress as well as lipid peroxidation and protein carbonylation in the cerebellar tissue of Sprague Dawley rats [27]. Previous research has demonstrated that brain tissue, particularly the cerebellum, is more susceptible to oxidative damage in DM conditions compared to other organs [27,28,29,30]. Although the role of oxidative stress in DM pathogenesis is well established, and this study confirmed the presence of oxidative and carbonyl damage in both diabetic groups, consistent with other studies [31,32], there were no differences in oxidative stress markers in the cerebellar tissue between the two phenotypically different ZDF fa/fa groups. This suggests a certain degree of resistance of cerebellar tissue to more advanced T2DM, despite more pronounced systemic oxidative stress indicated by differences in blood plasma between both ZDF fa/fa groups. In blood plasma, the difference in the severity of oxidative stress might result from the cumulative contributions of multiple tissues and organs, a common occurrence in type 2 diabetes. Previous reports have indicated that heart and renal tissues are not responsible for this difference [9,17]. Thus, when focusing on the working hypothesis, this study confirmed higher oxidative damage to the cerebellar tissue of ZDF rats compared with controls, although without differences dependent on DM severity.

**Figure 2:**
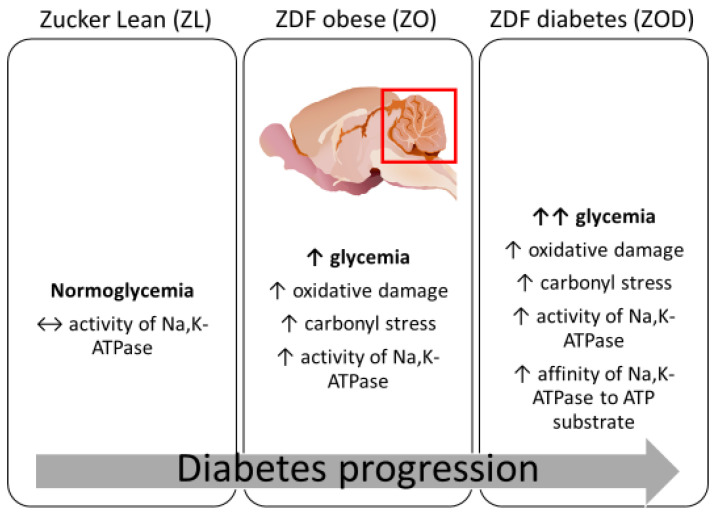
The schematic presentation of the key points of this study. Abbreviation: ZL—lean fa/+, Zucker diabetic fatty (ZDF) fa/fa rats divided into ZO rats with lower glycemia (<10 mmol·L^−1^) and ZOD rats with higher glycemia (>10 mmol·L^−1^), ↑—increase, ↑↑—greater increase, ↔—no change. The experimental animals were 38/39 weeks old.

The Na,K-ATPase enzyme is highly sensitive to changes in oxidative status [33,34,35,36]. In the vast majority of studies, Na,K-ATPase functionality is typically assessed through simple activity measurements under fixed conditions with a specific substrate concentration. However, such an approach lacks detailed information about the enzyme’s reaction dynamics. In this study, kinetic measurements of Na,K-ATPase enzyme activity were conducted at various substrate concentrations to provide a comprehensive and dynamic understanding of the enzyme’s activity and regulation. This approach allows for the determination of key parameters as such V_max_ (maximum reaction rate) and K_m_ (Michaelis constant). These parameters are crucial for characterizing enzyme functionality and comparing different physiological and pathophysiological conditions. V_max_ reflects the number of active enzyme molecules, while K_m_ indicates the enzyme affinity for binding the ATP substrate. Kinetic enzyme measurements can also provide insight into energy metabolism in the cerebellum of ZDF rats, which is influenced by obesity, hyperglycemia, and oxidative stress. Na,K-ATPase enzyme consumes approximately 50% of the brain’s available energy [37,38], making it a significant player in brain energy metabolism. Therefore, this study examined ATP utilization by Na,K-ATPase. The findings revealed that the ZOD group exhibited the highest Na,K-ATPase activity compared with all other groups, likely indicating an increase in the number of active enzyme molecules. Additionally, the lowered K_m_ value suggests an enhanced ability of the enzyme to bind its substrate (ATP). The key findings of the present study are illustrated in Figure 2.

It may be proposed that increases in lipid peroxidation, protein oxidation, and greater carbonyl stress would impair Na,K-ATPase activity. Indeed, this has been shown in the brains of the alloxan-induced diabetic model [39]; metabolic syndrome induced by a high-fat, high-sucrose diet combined with low doses of streptozotocin (STZ) [40]; and in the brain cortex of the STZ-induced diabetic model [41]. However, contrary to the working hypothesis, a higher number of active Na,K-ATPase molecules were observed in the cerebellum of ZDF fa/fa rats. The highest enzyme affinity for ATP was found in the ZOD rats compared with those with less advanced DM (the ZO group) and control Wistar rats. The difference in Na,K-ATPase activity between STZ-induced diabetic models and ZDF rats might be due to the direct inhibitory effect of STZ on this enzyme in brain tissue [42]. Additionally, insulin is known to stimulate Na,K-ATPase [43,44], and both groups of ZDF fa/fa rats in this study exhibited increased plasma insulin concentrations, which can at least partially cross the blood–brain barrier [45]. Another possible explanation for the increased Na,K-ATPase activity is the hypoactivity of the sympathetic nervous system at the central level in ZDF fa/fa rats [46], as Na,K-ATPase activity is inhibited by catecholamines [47,48]. In addition, the oxidative stress inducing the reduced production of the energy substrate ATP might also affect the properties of Na,K-ATPase in T2DM. Mitochondrial dysfunction and subsequent reduction in ATP synthesis were documented in 30–34-week-old ZDF rats [28]. The significantly lower K_m_ value in the ZOD group compared to the ZL group indicates better binding properties for ATP, especially at lower ATP concentrations, suggesting an adaptive response to reduced ATP levels due to simultaneous overweight and hyperglycemia. This aligns with a recent postmortem study of human hippocampal slices from elderly subjects over 80 years old with T2DM and vascular dementia, which proposed that reduced ATP production could be responsible for cerebral Na,K-ATPase dysfunction [49]. An increase in Na,K-ATPase activity could also represent a compensatory reaction to imbalance in calcium homeostasis frequently underlined in relation to diabetes [50,51]. Increases in intracellular calcium levels stimulate the Na,K-ATPase enzyme to keep the necessary sodium concentration gradient for calcium efflux via the sodium–calcium exchanger what was already observed in other brain disorders [52]. It should be added that, in the majority of studies involving human samples or animal models of neurodegeneration, Na,K-ATPase activity has been shown to be compromised in various tissues, including the nervous system [10,11,42,53,54,55]. Therefore, our findings of increased Na,K-ATPase activity do not support the occurrence of neurodegenerative processes in the cerebellar tissue of aged ZDF (fa/fa) animals.

Another aspect of this study examined differences between two commonly used control rat strains—lean ZDF fa/+ rats, standard controls for ZDF fa/fa rats, and Wistar rats—to highlight possible distinctions between wild-type rats and those heterozygous for a signaling-deficient receptor as suggested previously [56,57]. Despite significant variations in general characteristics between the Wistar and ZL groups, minimal differences in oxidative stress markers were observed in this study. The most significant finding was the increased affinity of Na,K-ATPase for ATP in heterozygotes compared with wild-type Wistar rats.

## 5. Conclusions

The results of this study confirmed significant differences in oxidative stress markers between diabetic and control rats, both at the systemic level and in cerebellar tissue. The extent of oxidative damage to lipids, proteins, and carbonyl compounds varied with the severity of diabetes (i.e., differences between ZO and ZOD groups), but these changes were only noticeable in the circulating markers. This implies that the cerebellar tissue might be somewhat resistant to the oxidative damage associated with the progression of T2DM, at least in the ZDF rat model of T2DM. Additionally, the Na,K-ATPase enzyme, which is known to be sensitive to oxidative damage, showed increased activity in the cerebellum of both diabetic groups (ZDF fa/fa) compared with controls. This may reflect a compensatory mechanism in aged ZDF (fa/fa) animals, rather than indicating cerebellar neurodegeneration: a phenomenon that warrants further investigation.

## Figures and Tables

**Table 1:** Basic biometric and biochemical characteristics of the laboratory rats.

	W (*n* = 12)	ZL (*n* = 12)	ZO (*n* = 8)	ZOD (*n* = 8)
BW (g)	488 ± 27	413 ± 10 *	628 ± 8 ****^,++++^	528 ± 17 ^+++,##^
Brain weight (g)	2.07 ± 0.02	1.96 ± 0.03 *	1.87 ± 0.03 ***	1.87 ± 0.02 ***
CW (g)	0.29 ± 0.009	0.25 ± 0.009 *	0.24 ± 0.011 **	0.24 ± 0.006 **
CW/BW × 10^5^	56.1 ± 1.0	61.1 ± 2.2	38.0 ± 2.0 ****^,++++^	45.3 ± 1.9 **^,++++^
Glucose (mmol·L^−1^)	7.2 ± 0.1	6.7 ± 0.1	8.8 ± 0.2 ^+^	18.3 ± 1.2 ****^,++++,####^
Insulin (ng·mL^−1^)	7.9 ± 1.8	1.6 ± 0.08 **	19.3 ± 2.1 ****^,++++^	8.7 ± 1.0 ^++,###^
HOMA-IR	2.77 ± 0.59	0.47 ± 0.02 **	7.49 ± 0.77 ****^,++++^	6.87 ± 0.63 ****^,++++^
HOMA-IS	20.55 ± 5.15	54.39 ± 2.75 ****	3.15 ± 0.24 *^,++++^	3.82 ± 0.36 *^,++++^
HOMA-β	1.18 ± 0.23	0.27 ± 0.02 **	2.51 ± 0.31 ***^,++++^	0.57 ± 0.09 ^####^

Abbreviations: W—Wistar, ZL—ZDF lean fa/+, Zucker diabetic fatty (ZDF) fa/fa rats divided into ZO rats with lower glycemia (<10 mmol·L^−1^) and ZOD rats with higher glycemia (>10 mmol·L^−1^), BW—body weight, CW—cerebellar weight, HOMA-IR—homeostatic model assessment for insulin resistance, HOMA-IS—homeostatic model assessment for insulin sensitivity, HOMA-β—homeostatic model assessment for β-cell function. Data are presented as means ± standard errors of mean (SEM). * *p* < 0.5, ** *p* < 0.01, *** *p* < 0.001, **** *p* < 0.0001 vs. W; ^+^ *p* < 0.05, ^++^ *p* < 0.01, ^+++^ *p* < 0.001, ^++++^ *p* < 0.0001 vs. ZL; ^##^ *p* < 0.01, ^###^ *p* < 0.001, ^####^ *p* < 0.0001 vs. ZO.

**Table 2:** Parameters of oxidative stress in plasma and cerebellum.

Cerebellum	W (*n* = 11–12)	ZL (*n* = 10–12)	ZO (*n* = 7–8)	ZOD (*n* = 7–8)
TBARSs (µmol·L^−1^)	34.20 ± 4.23	33.94 ± 3.57	60.17 ± 3.87 ***^,+++^	57.44 ± 2.33 ***^,+++^
AOPPs (µmol·L^−1^)	71.81 ± 7.74	89.74 ± 16.40	140.0 ± 10.07 **	127.2 ± 10.11 *
Fructosamine (mmol·L^−1^)	0.91 ± 0.14	0.87 ± 0.09	2.23 ± 0.52 **^,++^	1.37 ± 0.19
AGE-Fl (AU)	506 ± 8.22	489 ± 18.91	584 ± 9.09 **^,++^	613 ± 16.80 ****^,++++^
FRAP (µmol·L^−1^)	118.3 ± 17.12	228.3 ± 34.37 *	243.1 ± 35.16 *	176.7 ± 34.38
GSH/GSSG ratio	1.73 ± 0.05	1.60 ± 0.05	1.58 ± 0.11	1.44 ± 0.04 *
**Plasma**	**W (*n* = 15)**	**ZL (*n* = 15)**	**ZO (*n* = 7–8)**	**ZOD (*n* = 7–8)**
TBARSs (µmol·L^−1^)	10.00 ± 0.19	10.95 ± 0.23	20.69 ± 1.10 ****^,++++^	14.85 ± 1.13 ****^,+++,####^
AOPPs (µmol·L^−1^)	128.1 ± 9.53	286.8 ± 33.26	2135 ± 388 ****^,++++^	3427 ± 617.1 ****^,++++,#^
Fructosamine (mmol·L^−1^)	1.11 ± 0.05	1.42 ± 0.08	13.77 ± 2.27 ****^,++++^	7.64 ± 0.79 ****^,++++,###^
FRAP (µmol·L^−1^)	397.9 ± 19.63	473.5 ± 19.71	2917 ± 662 ****^,++++^	1617 ± 200 **^,++,##^
GSH/GSSG ratio	11.25 ± 0.55	11.13 ± 0.44	9.16 ± 0.17 *^,+^	9.77 ± 0.33

Abbreviations: W—Wistar, ZL—ZDF lean fa/+, Zucker diabetic fatty (ZDF) fa/fa rats divided into ZO rats with lower glycemia (<10 mmol·L^−1^) and ZOD rats with higher glycemia (>10 mmol·L^−1^), TBARSs—thiobarbituric acid reactive substances, AOPPs—advanced oxidation protein products, AGE-Fl—advanced glycation end product-associated fluorescence, AU—arbitrary units, FRAP—ferric reducing antioxidant power, GSH/GSSG—the reduced to oxidized glutathione ratio. Data are presented as means ± standard errors of mean (SEM). * *p* < 0.5, ** *p* < 0.01, *** *p* < 0.001, **** *p* < 0.0001 vs. W; ^+^ *p* < 0.05, ^++^ *p* < 0.01, ^+++^ *p* < 0.001, ^++++^ *p* < 0.0001 vs. ZL; ^#^ *p* < 0.5, ^##^ *p* < 0.01, ^###^ *p* < 0.001, ^####^ *p* < 0.0001 vs. ZO.

## Data Availability

The data that support the findings of this study are available in this article, while the raw data can be obtained from the corresponding author upon reasonable request.
